# Strategies for optimising health system managers’ engagement in quality improvement projects: lessons learnt from the COMPAS+ project

**DOI:** 10.1136/bmjoq-2025-003480

**Published:** 2025-12-04

**Authors:** Justin Gagnon, Brigitte Vachon, Mylaine Breton, Guylaine Giasson, Isabelle Gaboury

**Affiliations:** 1 Department of Family Medicine, McGill University, Montreal, Quebec, Canada; 2 Lady Davis Institute for Medical Research, Jewish General Hospital, Montreal, Quebec, Canada; 3 School of Rehabilitation, Faculty of Medicine, Université de Montréal, Montreal, Quebec, Canada; 4 Department of Community Health Sciences, Faculty of Medicine and Health Sciences, Université de Sherbrooke, Sherbrooke, Quebec, Canada; 5 Department of Family Medicine and Emergency Medicine, Faculty of Medicine and Health Sciences, Université de Sherbrooke, Sherbrooke, Quebec, Canada

**Keywords:** Communication, Management, Chronic disease management, Healthcare quality improvement, Health services research

## Abstract

**Background:**

Quality improvement strategies are used in healthcare to enhance the quality, safety and efficiency of service delivery. While the involvement of managers is considered critical, their roles remain underdocumented. This study examines the roles of managers in COMPAS+, a quality improvement collaborative conceived to enhance chronic disease care in Quebec, Canada. It explores managers’ specific contributions to quality improvement projects to deepen understanding of effective managerial engagement.

**Methods:**

This qualitative case study compares the roles played by managers (health network directors, division managers and local service network and family medicine group directors) within four regional health networks that participated in COMPAS+ from 2016 to 2019. Deductive and inductive thematic analysis of workshop reports, action plans and interviews with 24 key actors was performed, informed by a recent scoping review of decision-makers’ roles in quality improvement projects and project management literature.

**Results:**

The study revealed variability in project management across cases, particularly in the distribution of responsibility among upper, middle and lower management. Upper management provided strategic direction, middle management oversaw project execution and bridged organisational tiers, while lower management coordinated local change efforts. Middle managers were tasked with project management but often lacked role clarity and training. A significant gap was found in methodological guidance, typically provided by a quality improvement facilitator. This gap hindered projects’ potential and, in some cases, led to deviations from the intended quality improvement model.

**Conclusions:**

Effective quality improvement project management requires well-defined managerial roles, training and communication between management levels. Our findings highlight the importance of integrating a facilitator role to provide methodological expertise and ensure adherence to quality improvement processes. Contextual expertise and local change leadership may be complemented by external quality improvement expertise. These insights lay the groundwork for future research on evidence-based strategies for effective project management.

**WHAT IS ALREADY KNOWN ON THIS TOPIC**

- Managers are pivotal to quality improvement (QI) success, but their level‐specific functions and contributions within QI projects are underdocumented.

**WHAT THIS STUDY ADDS**

- Empirical, cross-case evidence from COMPAS+ maps the distribution of responsibilities across managerial tiers.
- Middle managers were typically tasked with project management without clear role definitions, protected time or QI-method training.

**HOW THIS STUDY MIGHT AFFECT RESEARCH, PRACTICE OR POLICY**

- This study highlights the need to explicitly define and resource a facilitation function matched to scope: internal managers with protected time and QI skills for smaller projects, and a hybrid model (external QI coach with an internal lead) for larger, multisite initiatives.
- Future research should compare facilitation configurations and examine the competencies, training needs and overall impact of the QI facilitator role in healthcare.

## Background

 Over the past decade, healthcare organisations have increasingly adopted quality improvement (QI) strategies to enhance service quality, safety and efficiency.^1 2^ Despite expectations, evidence of their impact has been mixed,^3^ with some studies reporting significant improvements and others showing limited or unsustained effects.^4^,^7^ The literature underscores the critical role of managers in the success of QI projects.^8^,^11^ Managers play a role in securing buy-in from frontline staff and in fostering a culture of continuous improvement.^12^,^15^ They also facilitate the alignment of QI projects with organisational strategies, ensure adequate resource allocation and enhance communication across managerial levels, thereby supporting the effective implementation and sustainability of QI efforts.^10 12 15 16^ Research suggests that projects in which managers play a prominent role are more likely to achieve their objectives and sustain improvements.^13 15 17 18^

Theoretical guidance regarding the roles of QI team members can be found in various project management frameworks. For instance, the Team Handbook^19^ delineates roles that are integral to successful QI projects: coach, team leaders, sponsors and team members. According to this framework, coaches facilitate team processes by guiding the application of QI methodologies, ensuring process adherence, facilitating problem-solving and nurturing effective team dynamics. Team leaders manage day-to-day operations, coordinate team activities and maintain communication among team members. Sponsors provide oversight and resources to ensure alignment with organisational goals. Finally, team members contribute knowledge and experience throughout the process and carry out hands-on tasks including data collection, analysis and implementation. Theoretical literature also highlights the importance of having clearly defined roles within QI teams to enhance team effectiveness, project coherence and sustainability.^20 21^ Evidence suggests that among these roles, the QI coach is particularly critical, not only in ensuring methodological fidelity, but also in fostering organisational capacity for sustained change.^22^

Despite the abundance of theoretical literature on QI project management, and assertions regarding the benefits of managerial engagement, there remains a notable lack of empirical research detailing the specific functions managers perform when actively involved in QI projects. As reported in a recent scoping review examining decision-makers’ roles in QI projects, managers’ specific contributions are typically undocumented.^11^ Evidence-informed guidance regarding managerial roles is therefore limited.^12 23 24^ Consequently, a gap persists in understanding not only what these roles look like in practice, but also the conditions that influence their effective engagement.

Our research addresses the gap between QI theory and practice by examining the roles played by managers involved in the COMPAS+ project, a large-scale QI collaborative (QIC) that aimed to enhance chronic disease care in Quebec.^25^ For the purposes of this study, ‘managers’ refers to organisational leaders in standing posts, such as regional health network directors, division managers and local service network (LSN) and family medicine group (FMG) directors. It does not include externally hired or dedicated project management professionals. The COMPAS+ project provides a suitable context for examining and identifying variations in managers’ roles throughout the organisational hierarchy and between regions. COMPAS+ offers a range of contextual conditions and role configurations for analysis, which enriches our understanding of how managerial engagement in QI varies across settings. The purpose of this research is to translate theoretical frameworks into actionable insights to support the more effective design and implementation of QI projects within healthcare organisations, ultimately increasing their likelihood of success. Specifically, this study examines the different roles these managers played in the COMPAS+ project, providing insights into how managerial engagement can be optimised to improve project effectiveness and outcomes.

COMPAS+ was initiated by the Quebec Ministry of Health and the Institut national d’excellence en santé et en services sociaux (INESSS). Through this initiative, regional health networks received structured support to lead QI projects that aimed to enhance the quality of primary care for diabetes and COPD management.^25 26^ COMPAS+ comprised collaborative activities coordinated by INESSS and was structured into three phases: preparatory, reflective workshop and follow-up. The preparatory phase involved selecting a target chronic condition, securing support from regional health network CEOs and directors, setting up local implementation committees and recruiting workshop participants. The QI workshop phase included reviewing administrative data and insights from population health analyses, engaging in facilitated group reflection and root cause analysis, and developing QI action plans. In the follow-up facilitation phase, each regional network was expected to designate a local project lead to coordinate activities with the INESSS facilitator. This phase spanned 24 months and comprised regular meetings between the COMPAS+ programme facilitator and each regional implementation committee to refine their action plans, provide support and monitor implementation progress. Although small-scale improvement cycles were initially planned, they were not consistently employed in each of the regions.

## Methods

### Study design

The present research comprises a comparative case study design. This study was embedded within a broader COMPAS+ mixed methods multiple case study research project (detailed in previous publications^25 27^), which aimed to assess the outcomes of the COMPAS+QIC. The cases examined in the present research comprise four Quebec regional health networks that participated in COMPAS+ between 2016 and 2019. While INESSS’s approach to engaging the regional health networks and the facilitation support provided were consistent across cases, each case adapted its project processes to local conditions and differed in its utilisation of the support provided. We anticipated that comparing these cases would provide insights into the range of managerial roles and a deeper understanding of the factors contributing to these differences.

### Data collection

For the present study, data were obtained from three sources: semistructured interviews (n=24) conducted in 2021–2022 with clinicians and regional chronic disease care managers; (2) COMPAS+ workshop reports and regional A3 action plans and (3) follow-up notes from the INESSS facilitator. Four to seven informants per case were purposively selected to maximise variation in management level (upper, middle, lower). Actors with first-hand involvement in COMPAS+ planning, coordination and follow-up were prioritised to ensure a credible account of decisions and actions surrounding project management. We judged this sample sufficient to achieve thematic saturation given the depth and relevance of participants’ experience. The interview guide was informed by Brennan’s Informing Quality Improvement Research in primary care framework,^28 29^ the Integrated Performance Management System,^30^ QI programme theory developed for COMPAS+^31^ and the Montreal Model of Patient Engagement.^32^ A trained research professional (GG) conducted the interviews, which were audio-recorded and transcribed to facilitate coding. All documents (reports, action plans and INESSS facilitator notes) were maintained electronically by the regional networks and INESSS and shared with the COMPAS+ research team.

### Data analysis

Our research, which focuses specifically on managerial roles, extends the broader mixed-methods research project through a secondary analysis of the qualitative data sources. Inductive-deductive thematic analysis was performed to extract themes related to the managers’ involvement. We developed an initial framework and coding grid by drawing on a recent scoping review of healthcare decision-makers’ roles in QI^11^ and the Team Handbook team roles taxonomy (coach, team leader, sponsor, team member),^19^ discussed above. Our framework supported our characterisation of different managerial contributions throughout the QI project lifecycle (planning, analysis, action plan, testing, implementation) by functional domain (strategic, tactical, operational).

The coding grid was iteratively refined. We piloted it on a sample of interviews, then the team reviewed outputs and refined themes and categories until a stable version was reached. Once the grid was finalised, we applied it to all interview transcripts and documentary sources (workshop reports, regional action plans, facilitator follow-up notes). To support comparison, we built actor-by-phase matrices for each case. Actors were assigned a managerial level based on their formal position, and their specific functions were coded interpretively against categories from the scoping review and the Team Handbook. We classified regional health network high-level directors as upper management, regional division directors as middle management, and LSN and FMG directors and managers as lower management. We synthesised patterns within cases and then conducted cross-case pattern matching, producing comparative tables from the matrices. Relevant passages were then extracted and compiled into comparative tables to support inter-network analysis. Regular exchanges between the researchers (JG, BV, IG) enabled triangulation across data sources (interviews, follow-up reports, action plans), and the project’s historical context.

Reflexivity was a key component of our methodological process. Two members of our research team (IG, BV) were closely involved in the COMPAS+ implementation, and two (JG, MB) served as neutral interpreters, uninfluenced by the project’s history. This combination enabled both a nuanced understanding of the project context and a critical, independent interpretation of the data. We recognise that our interpretations were inevitably influenced by our prior assumptions that managers hold critical responsibilities across strategic, tactical and operational levels. These presuppositions shaped our emphasis on role clarity and engagement as key contributors to project success. By explicitly acknowledging these assumptions, we aimed to increase transparency and reinforce the credibility of our analysis.

## Results

Regional characteristics and specifics of the QI projects conducted across the four cases are detailed in table 1. Cases A and D prioritised chronic obstructive pulmonary disease (COPD) care, whereas cases B and C focused on diabetes. A common aim, among proposed improvements, was to standardise care throughout the region, though strategies diverged. Cases A and B concentrated on developing standardisable tools and care pathways within a single LSN, with the intention of testing and subsequently scaling them region-wide. Case D pursued locally adapted solutions by implementing separate QI initiatives within each LSN and disseminating successful practices across the region. While case C also aimed to standardise care trajectories and educational materials, it focused instead on consolidating services through the creation of a regional centre of expertise in diabetes care.

**Table 1:** Comparison of proposed improvements by case

Case	Case A	Case B	Case C	Case D
Target condition	COPD	Diabetes	Diabetes	COPD
Proposed improvements				
Strategic function	Develop a standardised trajectory for the region	Develop a standardised trajectory for the region	Develop a standardised trajectory for the region Harmonise services and redirect acute cases to centre of expertise	Develop locally adapted trajectories and share findings with other LSNs
Tactical function	Improve screening and diagnostic processes Promote health system user education	Optimise chronic disease centre service mandates Enhance communication and coordination	Develop education and training programmes	Enhance primary care COPD screening and follow-up processes Enhance communication and coordination Integrate respiratory therapist
Operational function	Enhance and formalise clinical resources and guidelines Provide COPD education and training for healthcare providers	Formalise role definitions and responsibilities Enhance communication tools and processes	Provide education and training programmes for primary care nurses	Formalise role definitions and responsibilities Implement personalised COPD patient action plans Provide education and training programmes Increase awareness of COPD in the population Integration of primary care and hospital information systems to enhance communication and coordination
Implementation and scaling strategy	Conduct QI in select LSNs and spread best practices across the network	Conduct QI in select LSNs and spread best practices across the network	Establish a centralised centre of expertise to drive and scale improvements across the network	Conduct localised QI cycles in each LSN and share best practices across the network
Project committee composition	Follow-up on chronic disease management: Regional director (1) Deputy director (1) Regional coordinators (2) Regional programme managers (2) Medical advisors (2) Clinicians (2)	Continuous improvement for diabetes pathway Regional director (1) Deputy director (1) Regional coordinators (2) Regional programme manager (1) Clinicians (3) Project leader (1)	Diabetes management and strategic planning: Regional director (1) Deputy director (1) Regional coordinators (2) Clinician manager (1) Clinician (1)	Regional follow-up for COPD: Deputy director (1) Regional coordinator (2) Regional programme manager (1) Unit directors (3)

COPD, Chronic obstructive pulmonary disease; LSNs, local service networks; QI, quality improvement.

The comparison of proposed improvements presented in table 1 reveals differences in how the cases emphasise strategic, tactical or operational improvements. These are categorised according to the stages of an integrated management system.^30^ Notably, cases A and B prioritised tactical-level improvements focused on screening, diagnostic processes and patient education within the health system. For example, case A focused on developing clinical resources like screening decision aids and educating health system users, whereas case B planned to optimise chronic disease centre service mandates and enhance communication and coordination. By contrast, case C targeted structural and strategic changes, predominantly through the establishment of a new centre of expertise. As for case D, it primarily emphasised operational improvements, such as enhancing primary care screening, promoting COPD awareness and implementing personalised COPD patient action plans. Despite these differences in focus, all committees were similarly composed, with members spanning upper, middle and lower management tiers and engaging in activities across strategic, tactical and operational domains.

### Managerial roles

Table 2 provides a comparison of managerial contributions across different QI project phases, organised by management level. Here, we present an amalgamated view of responsibilities across cases to illustrate how engagement differed by management level.

**Table 2:** Managerial roles and responsibilities across quality improvement phases

Phases	Upper management	Middle management	Lower management
Preparatory	Strategic direction: Developed strategic plans and provided project approval. Resource advocacy: Secured necessary approvals and resources. Strategic decisions: Choose focus areas based on readiness and existing initiatives.	Organisational coordination: Managed logistical planning and resource alignment. Patient partner management: Recruited and coordinated with patient partners for workshop participation. Strategic planning: Engaged in planning sessions to align with healthcare priorities.	Operational planning: Coordinated logistics for workshop preparations. Local needs assessment: Identified specific regional healthcare challenges. Initial stakeholder engagement: Facilitated early discussions to define project scope, and engage participating clinics
Analysis	Strategic committee Involvement: Influenced workshop focus and planning. Advocacy for data-driven decisions: Promoted the use of data to guide improvements.	Data analysis and communication: Managed project documentation and facilitated stakeholder engagement. Consultation: Provided strategic and operational insights Workshop facilitation: Led and ensured active participation and fostered collaboration.	Active workshop participation: Provided insights and feedback to align goals with operational realities. Data collection coordination: Oversaw local health data collection and analysis. Advocacy and support: Ensured analytics addressed clinical needs and challenges.
Action plan	Action plan oversight: Managed the strategic planning and execution of action plans.	Task integration and operational supervision: Translated strategic plans into operational tasks and oversaw implementation. Resource management: Allocated and managed resources effectively.	Strategy formulation: Worked with frontline staff to develop action plans Action plan execution: Implemented action plans within local settings. Implementation coordination: Engaged frontline staff.
Testing	Evaluation of engagement: Monitored and refined strategies for engaging medical professionals with health data.	Performance monitoring: Oversaw project execution and adaptation based on outcomes. Follow-up and evaluation: Managed the integration of action plans and monitored their effectiveness.	Pilot testing and feedback: Conducted tests of proposed solutions and established feedback mechanisms to refine interventions.
Implementation	Implementation leadership: Drove broader adoption and integration of project outcomes. Support and governance: Ensured continued strategic support and oversight.	Implementation support and advocacy: Emphasised the need for ongoing support and resources. Planning for sustainability: Provided strategic support to ensure the alignment with broader organisational goals.	Operational execution and sustainability: Managed daily implementation and developed mechanisms for long-term project sustainability.

Our analysis displays a stratified approach to QI project management, with distinct roles assigned to different managerial levels with variable degrees of engagement in individual activities. Upper management predominantly orchestrated the strategic facets of the action plan. This included setting the overarching strategic direction and securing necessary resources during the preparatory phase. They also oversaw the broad implementation strategies during subsequent phases. In the initial phases, the middle managers played a key role in strategic planning, resource alignment, workshop coordination. During implementation, they monitored project progress, provided operational direction and support, and allocated resources. Lower management, on the other hand, was more involved in the operational aspects of the QI projects. Their responsibilities centred on logistical coordination, local needs assessments, action plan implementation and management of daily operational tasks.

Participants described considerable variability across regions in how these roles were enacted. The nature of the targeted changes also varied. Some regions, influenced by the strategic focus of their managers, adopted approaches resembling the Baldrige Framework,^33^ which emphasises broad organisational redesign and strategic planning over immediate operational changes. While this approach can yield long-term benefits, such as wider system alignment and performance management, it lacks the adaptability and rapid implementation focus of iterative QI models. As a result, these regions struggled to translate their strategic plans into concrete implementation strategies.

In the absence of clear role definitions, structured support and methodological expertise, some regions (eg, cases A and B) deviated from the intended QI approach. As a participant from case D explained, appointing a project lead (assign a QI project-management function to a manager occupying a standing post) was imposed as a condition for receiving support, but the managers often lacked the experience or capacity to fulfil this role.

As for the INESSS approach itself… there were places that didn't necessarily have a project manager, or it would be like saying “look, do you want us to get involved? Fine, but we are literally requiring you to appoint a project manager…” otherwise, it risks being just a pointless effort.

Another informant from case C noted that in their region, the service managers assumed the responsibility for project management, despite already being overburdened.

It was the service managers who did the work… They were directly responsible. And I can’t fault them because they already had too much on their plate. They did what they could to move things forward.

Ultimately, while strategic planning efforts were made in the initial phases, it often lacked the operational follow-through needed to implement changes on the ground. Instead of engaging in iterative improvement cycles, many regions focused on articulating high-level plans without a clear pathway for execution. This gap underscores the need to better define and support managerial responsibilities so that strategic intentions lead to tangible outcomes.

### Collaboration between managerial levels

**Figure 1:**
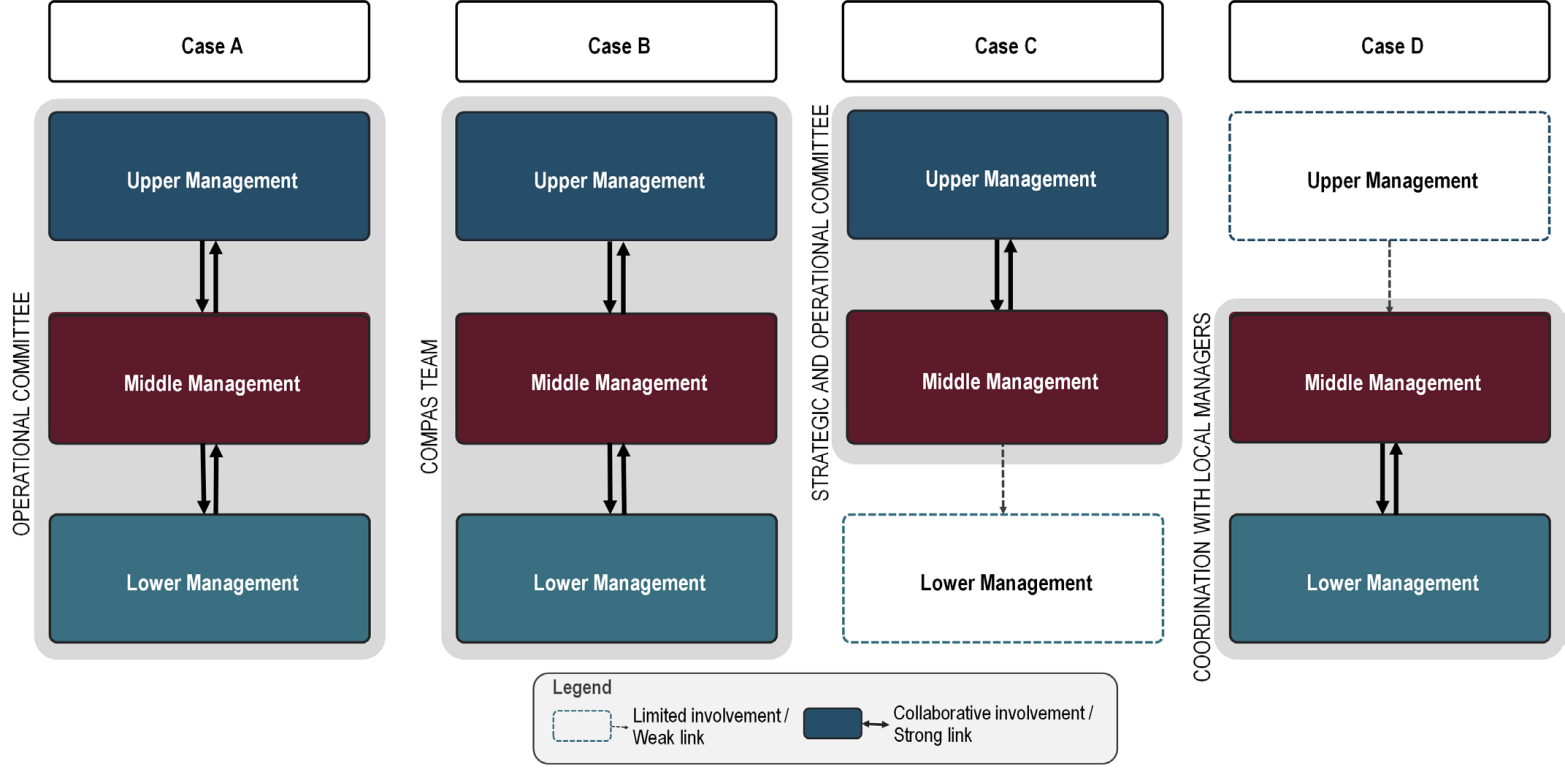
Regional comparison of relationship strength between managerial levels concerning COMPAS+.

Drawing on descriptions of committee composition from the regional networks’ action plans and reports from interview subjects, we developed a characterisation of the degree of collaboration between managerial tiers, displayed in figure 1. We then examined this characterisation in juxtaposition with the changes proposed by the regions in their action plans, summarised in table 1. This comparison allowed us to interpret how the involvement of different managerial levels shaped project aims, with different degrees of emphasis on strategic, tactical and operational change.

In case C, their project committee comprised primarily upper and middle managerial layers. Reflecting a strong influence from the strategic directorate, their aims included the creation of a centralised centre of expertise, the harmonisation of services, and the development and delivery of educational programmes for health professionals. In contrast, case D employed a more decentralised approach, with lower management (LSN and FMG directors) coordinating with the regional middle managers, emphasising greater collaboration among lower managerial levels, with less involvement of upper management. They focused more on tactical and operational aims, such as improved screening processes, enhanced communication, role clarification and promotion within the population. In cases A and B, since project committees included members from all managerial levels, we concluded that collaboration between managerial tiers was strong. In contrast with cases C and D, their action plans comprised a relatively balanced distribution of strategic, tactical and operational aims. This distinction reflects how managerial engagement influences both the scope and nature of proposed improvements.

A manager from case D emphasised the limitations of centralised, top-down approaches, stressing the need to adapt strategies to local contexts.

It absolutely has to come from the top down… but personally, I don’t believe in that… Yes, we can give broad orientations and guidelines, but we really need to adapt it from one place to another.

This perspective underscores the importance of active engagement and effective communication across all managerial levels in large-scale regional QICs. Gaps in communication, especially when operational activities are not aligned with broader organisational goals, can undermine project coherence and support. As one manager from case A noted, “I have seen other regions face issues because they were poorly connected with the executive committees… it was inadequately aligned with the strategic level.” Another informant described the consequences of infrequent communication: “We communicate when there’s a problem… otherwise, we don't communicate; it’s still very individualized… the communication doesn’t flow down.”

Middle managers, positioned between strategic leadership and frontline operations, are well-placed to bridge these gaps by ensuring that strategic directives address the realities of health service delivery, advocating for frontline staff and fostering collaboration across tiers to support smoother project execution. At the same time, as suggested by a manager in case D, sustained commitment from upper management remains critical: “The support… making sure that the COMPAS project is seen as a priority, with rigorous follow-up at that level and dedicated resources for it.”

In addition to their formal organisational roles, middle managers’ effectiveness was shaped by their ability to advocate for frontline staff and bridge strategic priorities with operational realities. Their impact is further strengthened by their experience, influence and the quality of their relationships with other stakeholders. As one participant observed, these individual qualities can make a significant difference in driving project progress and ensuring success.

Then we see, from discussing with several CISSS [regional health network] leaders, the project leaders… I realize that it’s really the individual personality of the leader, their experience, their vision, all of that. It seems to be really the key, the major factor for the advancement of the projects.

### The need for QI coaching and methodological support

In all four of the cases, the designated person responsible for coordinating the project within their region and liaising with the INESSS facilitator was a middle manager. Their contributions, as documented in table 3, included communication, stakeholder engagement and workshop facilitation during the initial phases. Following the workshops, they advocated for necessary resources, integrated strategic insights into operational tasks, monitored progress and assisted with the evaluation and dissemination of project outcomes.

**Table 3:** Comparison of middle manager roles and responsibilities

Phases	Case A	Case B	Case C	Case D
Preparatory	Communication and information dissemination Coordination and project oversight Initial stakeholder engagement	Organisation coordination Resource alignment Strategic planning	Organisation coordination Strategic planning	Local needs assessment Initial stakeholder engagement
Analysis	Workshop facilitation	Workshop facilitation	Workshop facilitation	Data collection coordination Planning of QI projects using results of workshops
Action plan	Action plan execution	Task integration and operational oversight Resource management	None reported	Action plan execution
Testing	Pilot testing and monitoring Follow-up and evaluation	Pilot testing and monitoring Follow-up and evaluation	None reported	None reported
Implementation	Implementation support Sustainability advocacy	Implementation support Sustainability advocacy	None reported	Implementation support

QI, quality improvement.

Although middle managers assumed responsibility for project management, many were not formally trained in QI and often lacked the methodological expertise required to maintain process fidelity. Referring again to the Team Handbook,^19^ we observed that the middle managers’ contributions aligned more closely with a team leader role, with their responsibilities including oversight of daily logistics, handling administrative tasks and facilitating team communication. Notably, no one was explicitly assigned the team role of ‘coach’, a position that typically involves guiding teams through QI methodologies, fostering team cohesion and supporting problem-solving.^19^ This absence limited the regions’ capacity to effectively design, implement and sustain QI projects. The COMPAS+ facilitator accompanied and supported regional managers, but they were not responsible for managing projects directly or offering comprehensive methodological guidance. As one participant from case D explained, managers were left to manage the COMPAS+project largely on their own: “You had a facilitator, but it was as if you had taken the project into your own hands […] you kind of took it over by yourselves without INESSS support.”

The middle managers also lacked the capacity to engage fully in the daily tasks central to continuous improvement, often due to their pre-existing organisational responsibilities. Another informant from case D expressed that a dedicated coordinator or facilitator would have been a critical asset.

Service managers don’t have the time to do it themselves. They have far too many things to handle. So, it really takes someone—a facilitator—to ensure that the process stays on track, that we continue to measure it, and that we… you know, it’s a continuous improvement process, so if there’s no follow-up, no evaluation, no feedback with the teams… Well, it’s pointless […] You need this kind of person.

The absence of a role encompassing methodological support and the daily tasks involved in iterative improvement emerged as a gap in the COMPAS+ project planning. This role, if clearly defined and well-supported, filled by individuals trained specifically in QI practices, would help to ensure adherence to QI processes and enable managers to focus more on strategic oversight. Cultivating these kinds of facilitative roles within QI initiatives presents a significant opportunity for healthcare organisations to bridge strategic priorities with continuous improvement processes, ultimately enhancing project outcomes and establishing the conditions for more sustainable impact.

## Discussion

### Statement of principal findings

This case study examines the roles of regional managers within COMPAS+, a regional QIC project. We stratified responsibilities across upper, middle and lower management. Upper management typically provided strategic direction and secured resources, particularly during initial phases. Middle management bridged strategy and operations by translating goals into actionable plans and brokered communication across tiers. Lower management coordinated local change efforts and led day-to-day implementation.

We observed an association between the dominant managerial tier on the project committee and the nature of the solutions proposed. Where upper management predominated (case C), action plans emphasised structural reorganisation. By contrast, where lower and middle management were more central (case D), plans emphasised tactical and operational changes. Cases with stronger cross-level collaboration (A and B) showed a more balanced mix of strategic, tactical and operational aims.

Our analysis also revealed considerable variability in how COMPAS+ was planned and managed across cases. A key factor was the implicit delegation of QI project management to middle managers, whose roles were often ill-defined and misaligned with their typical responsibilities. This was compounded by the absence of a dedicated facilitator or ‘coach’ to provide hands-on QI support and maintain process adherence. There also appeared to be an implicit assumption that managers would possess foundational competencies in project management, QI and change management. However, many appeared to lack these competencies, which differ markedly from the skillsets commonly associated with traditional administrative implementation. The COMPAS+ facilitator’s limited and sporadic involvement offered little support in bridging this gap. As a result, some regions drifted from adaptive, iterative QI towards more conventional, top-down approaches. These findings point to the need for QI training, clear role definitions, a supportive organisational culture, and an explicit facilitation function to safeguard methodological fidelity and strengthen cross-tier collaboration. In practice, this means reflecting on the scope, and available competencies and resources, and making an informed decision about whether this function ought to be fulfilled by a dedicated QI coach or by an existing manager with enhanced QI skills.

Our analysis also highlights the interplay between proposed improvements (strategic vs tactical vs operational), scaling strategies, the emphasis on specific managerial levels and cross-level collaboration. Leveraging this relationship is vital for designing and managing QI projects that effectively address both organisational and local needs. Directive top-down strategies, often met with resistance from frontline staff, can falter if strategic leaders lack insight into operational realities. Clear communication, precise role definitions and strong collaboration across all managerial levels are critical to bridging this gap. In large-scale regional QICs like COMPAS+, external facilitators bring added value by aligning initiatives across sites and ensuring effective project management.

### Interpretation in the context of the wider literature

This study contributes significantly to the growing body of research on the role of managers in QI initiatives, particularly within large-scale healthcare projects like the COMPAS+QIC. Consistent with the literature, our findings underscore the critical importance of managerial involvement at all organisational levels to achieve meaningful and sustained improvements.^8^,^1012 15 34^

The observed variability in managerial effectiveness highlights a persistent gap between the theoretical promise of QI projects and their practical outcomes.^3 7 35^ While previous studies emphasise the need for role clarity and cross-level collaboration,^12 16^ the specific contributions of managers throughout the QI lifecycle remain underdocumented, impeding the establishment of evidence-based guidelines.^11^ Our study helps fill this gap by providing empirical insights into the distinct contributions of managers across QI project phases.

The principles of the shared governance model, which emphasise decentralised decision-making, collaboration and shared accountability, offer a useful framework for bridging the gap between strategic vision and operational realities.^36 37^ Sustained engagement across managerial levels, combined with shared governance, equips organisations to address emerging challenges, align strategic goals with operational realities and maintain project momentum.^22^ These appear to be essential for enhancing the effectiveness and sustainability of QI initiatives.

A key insight from this study is the impact of gaps in managerial expertise and structured support on QI implementation. In the COMPAS+ project, the absence of role clarity and structured training left managers reliant on external facilitators, whose periodic involvement limited their ability to provide consistent support. This gap in expertise underscores the need for comprehensive QI support and to better equip managers for facilitative roles, enabling sustained methodological adherence and more successful implementation initiatives.

### Implications for policy, practice and research

#### Policy implications

The findings from this study present important policy implications, emphasising the need for initiatives that not only equip managers with technical skills but also foster a shift toward supportive and empowering leadership. Policies should draw on frameworks such as learning organisations^38^ and shared governance,^37^ which emphasise collaboration, shared accountability and decentralised decision-making, to better prepare managers to navigate the complexities of QI initiatives.

To address gaps in QI expertise, policies should allocate resources to establish and support dedicated facilitative roles, while providing clear guidelines to help managers align their efforts with QI objectives. The observed deviations from the intended QI model, which would have involved multiple rapid iterations, highlight the importance of also equipping senior management with the knowledge and tools necessary to provide strategic support and maintain alignment with QI principles.

#### Practice implications

The varied outcomes across regions in the COMPAS+ project illustrate the complex relationship between management structure, role clarity and effective inter-level communication. A hybrid model may have been more effective, balancing centralised coordination for knowledge sharing and prioritisation with decentralised approaches enabling the adaptation of QI strategies to regional needs.

Facilitators are central to this model, bridging gaps between strategic planning and operational realities. Evidence comparing external versus internal facilitation is mixed.^39^,^42^ External facilitators, such as those from INESSS, bring specialised methodological expertise, comparative benchmarks and perceived neutrality. They can challenge entrenched practices and mobilise teams to run rapid improvement cycles by introducing structured frameworks and measurement routines. However, external facilitators can face challenges building trust and integrating into organisational routines. Internal facilitators (ie, healthcare managers trained in QI), by contrast, offer deep contextual knowledge, established relationships and long-term engagement that support sustained change. Yet they may face role conflict, competing operational demands and variable depth in QI methods.

Facilitation configuration should be planned according to project scope and complexity. For smaller or more contained initiatives, internal managers with enhanced QI competencies may be able to integrate facilitation into their role if provided with adequate protected time and analytic support. For larger or more complex initiatives (eg, multisite, cross-sector projects), however, a hybrid structure may be more appropriate. External QI coaches may be leveraged to supply methodological discipline, sustain iterative improvement cycles and relieve operational managers of competing demands, while a designated internal project lead ensures contextual alignment, relationship management and local execution. This strategy balances external objectivity and internal credibility, enhances communication across tiers and helps align QI initiatives with both organisational priorities and local needs. Regardless of configuration, foundational requirements include a clear mandate with decision authority, defined competencies (QI methods, change management, facilitation), and adequate resourcing (eg, protected time and analytical support).

#### Research implications

The study identifies several areas for further research. Given the variability observed across cases, further investigation is needed to understand the organisational dynamics that support effective managerial involvement at different levels. In addition, more empirical work is warranted on the competencies, training needs and overall impact of the QI facilitator role in healthcare. This includes investigating under what conditions the role may be filled by a middle manager, when adequately trained and supported, and when a dedicated internal or external facilitator is more effective.

### Strengths and limitations

This study possesses several strengths that contribute to its value in the literature on managerial roles in QI initiatives. First, the study’s comparative case study design allowed for an in-depth exploration of managerial roles across multiple regions within the COMPAS+project, providing a rich, context-specific understanding of how managerial involvement can influence the outcomes of large-scale QI initiatives. The inclusion of various managerial levels in the analysis offered a comprehensive view of the interplay between strategic direction and operational execution, which is often underrepresented in QI literature.

However, the study also has limitations that should be acknowledged. One key limitation is that the research involved secondary analysis of data originally collected to interpret COMPAS+ outcomes and identify implementation barriers and facilitators. While managerial involvement was a significant focus, it was not the primary objective of the original data collection, potentially limiting the depth of insights specifically related to managerial roles. Despite this, the study serves as an introductory examination and offers valuable preliminary insights to guide more targeted future research.

Another limitation is the potential challenge in generalising the findings beyond the Quebec-based context of the COMPAS+ initiative. Managerial structures, resource availability and organisational cultures can vary significantly across different regions and healthcare systems, which may impact the transferability of the study’s conclusions to other settings, particularly single-site projects or those in different healthcare environments. However, this study does not propose a one-size-fits-all managerial strategy; instead, it offers a nuanced exploration of how various approaches can affect QI project outcomes. By providing detailed contextual descriptions and comparing different managerial roles and strategies, the study aims to equip readers with the tools to adapt these findings to their own contexts.

## Conclusions

This study underscores the importance of engaging managers across all levels to support the success of healthcare QI initiatives. The findings illustrate the necessity of formally defining roles, particularly the QI facilitator, who provides methodological guidance and ensures fidelity to QI principles. It seems imperative that managers at every tier understand and enact their specific responsibilities in fostering a culture of continuous QI. By formally integrating key roles into QI project management frameworks, and supporting them with training and adequate resources, healthcare organisations can strengthen their strategies and the culture that sustains them. These insights provide a solid foundation for future research to further refine evidence-based strategies, optimising QI project outcomes across varied healthcare contexts.

## Data Availability

No data are available.

## References

[1] MacGillivray TE (2020). Advancing the Culture of Patient Safety and Quality Improvement. Methodist Debakey Cardiovasc J.

[2] Cantiello J, Kitsantas P, Moncada S (2016). The evolution of quality improvement in healthcare: Patient-centered care and health information technology applications. *JHA*.

[3] Akmal A, Podgorodnichenko N, Foote J (2021). Why is Quality Improvement so Challenging? A Viable Systems Model Perspective to Understand the Frustrations of Healthcare Quality Improvement Managers. Health Policy.

[4] Wells S, Tamir O, Gray J (2018). Are quality improvement collaboratives effective? A systematic review. BMJ Qual Saf.

[5] Deblois S, Lepanto L (2016). Lean and Six Sigma in acute care: a systematic review of reviews. Int J Health Care Qual Assur.

[6] Knudsen SV, Laursen HVB, Johnsen SP (2019). Can quality improvement improve the quality of care? A systematic review of reported effects and methodological rigor in plan-do-study-act projects. BMC Health Serv Res.

[7] Endalamaw A, Khatri RB, Mengistu TS (2024). A scoping review of continuous quality improvement in healthcare system: conceptualization, models and tools, barriers and facilitators, and impact. BMC Health Serv Res.

[8] Gardner KL, Dowden M, Togni S (2010). Understanding uptake of continuous quality improvement in Indigenous primary health care: lessons from a multi-site case study of the Audit and Best Practice for Chronic Disease project. Implement Sci.

[9] Johns Hopkins Medicine (2022). Quality improvement: johns hopkins medicine. https://www.hopkinsmedicine.org/nursing/center-nursing-inquiry/nursing-inquiry/quality-improvement.html#7.

[10] Zjadewicz K, White D, Bouchal SR (2016). Middle managers’ role in quality improvement project implementation, are we all on the same page? – A review of current literature. *Saf Health*.

[11] Gagnon J, Breton M, Gaboury I (2024). Decision-maker roles in healthcare quality improvement projects: a scoping review. BMJ Open Qual.

[12] Pannick S, Sevdalis N, Athanasiou T (2016). Beyond clinical engagement: a pragmatic model for quality improvement interventions, aligning clinical and managerial priorities. BMJ Qual Saf.

[13] Parand A, Dopson S, Vincent C (2013). The role of chief executive officers in a quality improvement: a qualitative study. BMJ Open.

[14] Vaughn T, Koepke M, Kroch E (2006). Engagement of Leadership in Quality Improvement Initiatives: Executive Quality Improvement Survey Results. J Patient Saf.

[15] Mousavi Isfahani H, Tourani S, Seyedin H (2019). Features and Results of Conducted Studies Using a Lean Management Approach in Emergency Department in Hospital: A Systematic Review. Bull Emerg Trauma.

[16] Kirchner JE, Parker LE, Bonner LM (2012). Roles of managers, frontline staff and local champions, in implementing quality improvement: stakeholders’ perspectives. J Eval Clin Pract.

[17] Nyström ME (2009). Characteristics of Health Care Organizations Associated With Learning and Development. Qual Manag Health Care.

[18] Parand A, Dopson S, Renz A (2014). The role of hospital managers in quality and patient safety: a systematic review. BMJ Open.

[19] Scholtes PR, Joiner BL, Streibel BJ (2003). The team handbook: oriel.

[20] Lising D, Sinclair L, Rowland P (2021). Translating concepts to practice: Examining the synergy of interprofessional competencies and quality improvement. J Interprof Educ Pract.

[21] Hart CK, Dykes C, Thienprayoon R (2015). Change Management in Quality Improvement: The Softer Skills. Curr Treat Options Peds.

[22] Ballengee LA, Rushton S, Lewinski AA (2022). Effectiveness of Quality Improvement Coaching on Process Outcomes in Health Care Settings: A Systematic Review. J Gen Intern Med.

[23] Dainty KN, Sinclair D (2017). A Critical Qualitative Study of the Position of Middle Managers in Health Care Quality Improvement. J Nurs Care Qual.

[24] Nadeem E, Olin SS, Hill LC (2013). Understanding the components of quality improvement collaboratives: a systematic literature review. Milbank Q.

[25] Vachon B, Gaboury I, Menear M (2020). Evaluating implementation and impact of a provincial quality improvement collaborative for the management of chronic diseases in primary care: the COMPAS+ study protocol. BMC Fam Pract.

[26] Pomey M-P, Menear M, Drouin C (2020). Amélioration des soins et des services en première ligne pour la gestion des maladies chroniques : le programme COMPAS+ au Québec. Revue Française Des Affaires Sociales.

[27] Vachon B, Giasson G, Gaboury I (2022). Challenges and Strategies for Improving COPD Primary Care Services in Quebec: Results of the Experience of the COMPAS+ Quality Improvement Collaborative. Int J Chron Obstruct Pulmon Dis.

[28] Brennan SE, Bosch M, Buchan H (2012). Measuring organizational and individual factors thought to influence the success of quality improvement in primary care: a systematic review of instruments. Implement Sci.

[29] Brennan SE, Bosch M, Buchan H (2013). Measuring team factors thought to influence the success of quality improvement in primary care: a systematic review of instruments. Implement Sci.

[30] Moisan L, Fournier P-L, Lagacé D (2021). The Integrated Performance Management System: A Key to Service Trajectory Integration. Int J Integr Care.

[31] Vachon B, Désorcy B, Camirand M (2013). Engaging primary care practitioners in quality improvement: making explicit the program theory of an interprofessional education intervention. BMC Health Serv Res.

[32] Pomey M-P, Flora L, Karazivan P (2015). The Montreal model: the challenges of a partnership relationship between patients and healthcare professionals. Sante Publique.

[33] National Institute of Standards and Technology (2023). Baldrige excellence framework (health care): proven leadership and management practices for high performance. 2023–2024 edition ed.

[34] Maccoby M, Norman CL, Norman CJ (2013). Transforming health care leadership: a systems guide to improve patient care, decrease costs, and improve population health.

[35] Limato R, Tumbelaka P, Ahmed R (2019). What factors do make quality improvement work in primary health care? Experiences of maternal health quality improvement teams in three Puskesmas in Indonesia. PLoS One.

[36] Guanci R, Medeiros C (2025). Shared governance: what it is, and what it is not. https://chcm.com/shared-governance-what-it-is-and-what-it-is-not/.

[37] Clavelle JT, Porter O’Grady T, Weston MJ (2016). Evolution of Structural Empowerment: Moving From Shared to Professional Governance. J Nurs Adm.

[38] Smith M, Saunders R, Stuckhardt L (2013). Best care at lower cost: the path to continuously learning health care in America.

[39] Miller R, Weir C, Gulati S (2018). Transforming primary care: scoping review of research and practice. *J Integr Care (Brighton*).

[40] Ye J, Zhang R, Bannon JE (2020). Identifying Practice Facilitation Delays and Barriers in Primary Care Quality Improvement. J Am Board Fam Med.

[41] Parchman ML, Anderson ML, Coleman K (2019). Assessing quality improvement capacity in primary care practices. BMC Fam Pract.

[42] Ritchie MJ, Parker LE, Edlund CN (2017). Using implementation facilitation to foster clinical practice quality and adherence to evidence in challenged settings: a qualitative study. BMC Health Serv Res.

